# Metabolomic and proteomic analyses of renal function after liver transplantation

**DOI:** 10.3389/frtra.2025.1572852

**Published:** 2025-04-29

**Authors:** Xiaoling Wang, Nadja Grobe, Barbara Franchin, Josh Levitsky, Paolo Cravedi, Peter Kotanko

**Affiliations:** ^1^ Basic and Applied Laboratory Sciences, Renal Research Institute, New York, NY, United States; ^2^ Translational Transplant Research Center (TTRC), Department of Medicine, Icahn School of Medicine at Mount Sinai, New York, NY, United States; ^3^ Division of Gastroenterology and Hepatology, Comprehensive Transplant Center, Northwestern University Feinberg School of Medicine, Chicago, IL, United States

**Keywords:** liver transplantation, renal dysfunction, metabolomics, proteomics, mass spectrometry

## Abstract

**Background:**

Renal dysfunction is a common and serious complication in patients with end-stage liver diseases. While some patients recover renal function after liver transplantation (LT), others do not. Additionally, patients with normal kidney function (Normal-KF) before LT may develop post-transplant renal dysfunction. Early identification of patients at risk for impaired kidney function (Impaired-KF) post-LT is critical to improving outcomes. This study integrated metabolomic and proteomic analyses to investigate molecular profiles distinguishing Normal-KF from Impaired-KF post-LT.

**Methods:**

Nine LT recipients were classified into Normal-KF (*n* = 5) and Impaired-KF (*n* = 4) groups. One additional recipient with pre-transplant renal function impairment who recovered renal function after LT, was analyzed separately. Serum samples were collected at 2- and 5-weeks post-LT. The metabolomic and proteomic profiles were assessed by untargeted liquid chromatography-tandem mass spectrometry.

**Results:**

Metabolomic analysis identified 29 significantly altered metabolites between Normal-KF and Impaired-KF (fold change > 2, *p* < 0.05). Proteomic analysis revealed 45 differentially expressed proteins (fold change > 1.25, *p* < 0.05). For the recovered patient, the metabolomic profile closely resembled Normal-KF, whereas the proteomic profile remained aligned with Impaired-KF at both 14- and 35-days post-LT. From week 2 to week 5, both the metabolomic and proteomic profiles of the recovered patient showed trends toward the Normal-KF.

**Conclusion:**

This study revealed distinct metabolomic and proteomic signatures associated with renal dysfunction post-LT. Proteomic profiles indicated a delayed recovery compared to metabolomic profiles, suggesting a dynamic and muti-layered renal recovery process. Further research is warranted to elucidate the functional implications of the differential proteins and metabolites for improved monitoring and therapeutic strategies.

## Introduction

Liver transplantation (LT) is a life-saving intervention for patients with end-stage liver disease. However, renal dysfunction remains a common and serious complication that can occur both before and after LT (1–3). While some patients with kidney impairment prior to LT may recover their renal function post LT alone, others do not. Additionally, patients with normal kidney function (Normal-KF) before LT can develop post-transplant renal dysfunction, significantly impacting their long-term survival (4). Early identification of patients at risk for impaired kidney function (Impaired-KF) post-LT and the implementation of nephroprotective strategies are, therefore, critical to improving clinical outcomes in this vulnerable population (5).

Currently, the glomerular filtration rate (GFR), estimated using serum creatinine, is a widely used method in clinical practice for assessing renal function post-LT (6, 7). Although serum creatinine measurement is simple and cost-effective, it frequently overestimates kidney function in patients with liver disease, leading to delays in the diagnosis of renal dysfunction (2). This overestimation is likely due to decreased creatinine production and measurement interference from elevated bilirubin (3). Moreover, a single creatinine measurement provides only a limited perspective on renal function, overlooking its dynamic complexity and potentially obscuring the underlying causes of kidney injury.

To this extent, previous investigations have evaluated several proteins, such as Cystatin c (8), neutrophil gelatinase-associated lipocalin (NGAL) (9), beta-2-microglobulin (β2M) (10), tissue inhibitor of metalloproteinases 1 (TIMP-1) and osteopontin (OPN) (11, 12), as potential biomarkers for screening, diagnosing, and monitoring kidney function (13). In this study, we conducted an integrated metabolomics and proteomics analysis to gain deeper insight into kidney function in patients post-LT. By comparing patients with normal and impaired KF post-LT, we aimed to identify significant alterations in metabolites, proteins and their associated biological pathways. Additionally, we analyzed the molecular profile of a patient with recovered renal function to explore the timely changes associated with renal recovery.

## Methods

### Patients and samples

Ten patients from the multicenter, prospective study, NIAID CTOT14 (National Institute of Allergy and Infectious Diseases Clinical Trials in Organ Transplantation 14; NCT01672164) were included (14). Informed consent was obtained from all participants in accordance with IRB-approved protocols. All ten patients received LT. Two serum samples were collected from each patient at 2 weeks (mean 17 ± 4 days; range 13–24 days) and 5 weeks (mean 36 ± 15 days, range 20–66 days) post-LT. All samples were stored at −80°C until analysis.

### Metabolomics analysis

#### Sample preparation

Fifty microliter of serum were mixed with 11 µl of isotope-labeled metabolites from the QRess Kit (Cambridge Isotope Laboratories, MA, USA, catalog #MSK-QRESS-KIT, reconstituted per manufacturer's instructions). Metabolites were extracted with cold methanol at a final concentration of 72%. The mixture was vortexed and centrifuged at 4,303 × *g* for 15 min at 4°C. Seventy-five microliter of supernatants were transferred to a 96-well plate and dried using a cold-trap vacuum drier (Labconco®, MO, USA). The dried extracts were stored at −80°C and reconstituted on the day of analysis.

#### Chromatographic conditions

Five microliter of each reconstituted sample were injected into a reversed-phase liquid chromatography (LC) column (Phenomenex Luna C18, Part Number 00F-4114-E0), employing 0.01% formic acid and 1 mM ammonium formate in water as Solvent A and in methanol as Solvent B, with a flow rate of 0.75 ml/min. The initial condition was set at 1% B. The gradient was as follows: 0–0.5 min: 1% B; 0.5–7 min: linear gradient from 1% to 40% B; 7–8.5 min: linear gradient from 40% to 90% B; 8.5–12 min: linear gradient from 90% to 100% B; 12–12.5 min: 100% B; 12.5–13 min: linear gradient from 100% to 1% B; followed by equilibration at 1% B for 3 min.

#### Mass spectrometry (MS) and MS/MS data acquisition

MS data were acquired on Agilent Q-TOF 6546 equipped with a JetStream ElectroSpray Ionization source in both positive and negative ionization modes. Ion source conditions for positive mode were as follows: gas temperature 350°C, drying gas flow 12 L/min, nebulizer 35 psi, fragmentor 125 V, skimmer 65 V, and capillary voltage 4,000 V. For negative mode, ion source conditions were the same except fragmentor 51 V and capillary voltage −2,000 V. In addition to single injections of reconstituted sample, a pooled sample of all reconstituted extracts underwent the same LC procedure as described earlier. Following separation, MS/MS data of the pooled sample were acquired using iterative MS/MS.

#### Metabolites identification and quantification

MS/MS data were analyzed with MassHunter Qualitative Analysis software, matching results against both an in-house metabolite library (703 compounds with spectra and retention times) and the MassHunter METLIN library (Agilent Technologies, Santa Clara, CA). Metabolites identified via the in-house library were assigned ID confidence level 1, while those identified through the METLIN library, lacking retention time matches, were assigned ID confidence level 2 (15). The precursor ion of the identified metabolites and their corresponding retention times were compiled into a target list, which was used in Agilent Profinder to quantify the signal intensity of each metabolite across all LC-MS data from all samples.

#### Metabolomic data quantification criteria

A pooled sample of all reconstituted extracts was used as the quality control (QC). QC injections were performed at the start, after every 5 samples, and at the end of the sample injection. For each metabolite, the coefficient of variation (CV) was calculated based on 5 QCs. QC were also injected at 2-, 4-, 8-, and 16-fold dilutions, and *R*^2^ was determined by linear regression. Intensity data for each metabolite were filtered based on following criteria, including signal-to-noise ratio > 5, CV < 30% for QC, QC to QC-4-fold dilution ratio > 1.44, and *R*^2^ > 0.8 for the series-diluted QC.

#### Metabolomic statistical data analysis

The average peak intensities of each metabolite (measured at two time points from the same patient) were used. Identified drug compounds were excluded from the list. Data were Log2-transformed prior to analysis. Differential metabolites were identified using a Welch's *t*-test, with significance defined as a *p*-value < 0.05 and a fold change > 2. Principal component analysis (PCA) and hierarchical clustering heatmap were performed using web-based platform MetaboAnalyst 6.0 (https://www.metaboanalyst.ca/) (16).

### Proteomics analysis

#### Sample preparation

For each serum sample, 1.2 µl were processed using an EasyPep™ MS Sample Prep Kit (Thermo Fisher A40006) and resuspended in 80 µl of 0.1% formic acid. Additionally, 1 µl of each serum were pooled, treated with HSA/Immunoglobulin Depletion Columns (Thermo Fisher A36365), and processed using the same MS sample preparation protocol as the individual serum samples.

#### Chromatographic conditions

Each processed sample was separated by a reversed-phase LC column (Agilent AdvanceBio peptide mapping, Part Number 653750-902), employing 0.1% formic acid as Solvent A and 0.1% formic acid in acetonitrile as Solvent B, with a flow rate at 0.4 ml/min for a duration of 51 min. The gradient used for separation was as follows: 0–1 min: 3% B; 1–41 min: linear gradient from 3% to 45% of B; 41–44 min: linear gradient from 45% to 95% of B; 44–47 min: 95% B; 47–49 min: linear gradient from 95% to 3% B; 49–51 min: 3% B.

#### MS and MS/MS data acquisition

Following separation, LC-MS data were acquired on Agilent Q-TOF 6546 using auto MS mode. Ion source conditions were as follows: gas temperature 325°C, drying gas 12 L/min, nebulizer 35 psi, fragmentor 175 V, skimmer 65 V and capillary voltage 4,000 V.

Additionally, LC-MS/MS data was acquired from the pooled, HSA/Immunoglobulin-depleted sample using iterative MS/MS modes.

#### Peptide identification and quantification

The LC-MS/MS data were analyzed by Spectrum Mill software. The spectral data were searched against the reviewed proteins in the UniProt database. The identified peptides and their corresponding retention times were compiled into a target list, which was used in Agilent Profinder to quantify the signal intensity of each peptide across all LC-MS data.

#### Proteomic data quantification criteria

Pooled peptides from all samples were used as QC, with QC injections and analysis performed similarly to the metabolomic workflow. Quantification criteria were also identical, with the additional requirements of an MS score > 75 in Profinder and an MS2 score > 9 in Spectrum Mill.

#### Proteomic statistical data analysis

The average peak intensities of each peptide (measured at two time points from the same patient) were used. Statistical analysis was conducted at the protein level. For proteins with a single identified peptide meeting the quantification criteria, that peptide was used to represent the protein. For proteins with multiple identified peptides, a *p*-value (*t*-test) and fold change were calculated for each peptide. The median *p*-value and median fold change were then used to represent the protein. Significant proteins were defined as a *p*-value < 0.05 and a fold change > 1.25. For clustering analysis, each peptide intensity was normalized to its median intensity across all samples. For proteins with multiple peptides, the median normalized intensity was used to represent the protein. PCA and hierarchical clustering were performed using MetaboAnalyst 6.0.

## Results

### Study group

Ten patients with end-stage liver disease (numbered from 1 to 10) who underwent successful LT were included in this study (Table 1). GFR was estimated using the creatinine-based Chronic Kidney Disease Epidemiology Collaboration (CKD-EPI) formula (17). Of these, 5 patients (No. 1–5) with an eGFR greater than 60 ml/min/1.73 m^2^ both before and 2 weeks after LT were classified into the Normal-KF group. Four patients (No. 7–10) with eGFR less than 45 ml/min/1.73 m^2^ both before and 2 weeks after LT were assigned to Impaired-KF group. The baseline characteristics of patients at the time of LT were comparable between the two groups, and no significant differences in liver function were observed 2 weeks post-LT. The remaining patient (No. 6), with an eGFR of 23 ml/min/1.73 m^2^ before LT, showed an increase in eGFR to 76 ml/min/1.73 m^2^ 14 days post-LT and to 102 ml/min/1.73 m^2^ 35 days post-LT. This patient was designated as a recovered case.

**Table 1:** Patient characteristics.

Variable	Total (*N* = 10)	Normal-KF (*N* = 5)	Impaired-KF (*N* = 4)	Recovered (*N* = 1)	*p-*value** (normal-KF vs. impaired-KF)
Age, years	59.7 (6.9)	57.8 (4.7)	65 (5.1)	48	0.07
Female gender	4 (40%)	2 (40%)	2 (50%)	0	1
Cause of end-stage liver disease (ESLD)					
Non-alcoholic fatty liver or cryptogenic	3 (30%)	1 (20%)	2 (50%)	0	1
Hepatitis C (non-viremic)	5 (50%)	3 (60%)	2 (50%)	0	1
Alcohol	2 (20%)	1 (20%)	0 (0%)	1	1
ALT post-transplant W2 (U/L)	26.5 (13.7)	31.6 (16.4)	22.4 (11.0)	10	0.41
Alkaline phosphatase post-transplant Week 2 (U/L)	130.6 (49.7)	147.6 (52.0)	128 (91.7)	100	0.41
eGFR pre-transplant (ml/min/1.73 m^2^)*	60.5 (38.9)	90.6 (18.2)	23.1 (17.5)	23	0.005
eGFR post-transplant Week 2 (ml/min/1.73 m^2^)*	51.9 (32.0)	75.8 (6.9)	15.8 (10.8)	76.4	0.0002
eGFR post-transplant Week 5 (ml/min/1.73 m^2^)*	47.6 (34.8)	66.1 (14.3)	11.0 (6.5)	101.6	0.0003
Pre-transplant hypertension	3 (30%)	2 (40%)	0 (0%)	1	0.44
Pre-transplant diabetes	3 (30%)	2 (40%)	1 (25%)	0	1
Maintenance immunosuppression					
Tacrolimus	10 (100%)	5 (100%)	4 (100%)	1	1
MMF or MPA	10 (100%)	5 (100%)	4 (100%)	1	1
Steroid	8 (80%)	4 (80%)	4 (100%)	0	1
mTOR inhibitor	1 (10%)	0 (0%)	1 (25%)	0	0.44

Results are reported as Number (proportion) or Mean (standard deviation). Normal-KF, normal kidney function; Impaired-KF, impaired kidney function; eGFR, estimated glomerular filtration rate; ALT, alanine aminotransferase.

* GFR was estimated using the creatinine-based Chronic Kidney Disease Epidemiology Collaboration (CKD-EPI) formula.

** *p-*value is calculated using either Welch's *t*-test (continuous variables) or Fisher exact test (categorical variables).

### Metabolomic analysis

A total of 138 identified compounds met the quantification criteria. Of these, 30 compounds were medications, such as Tylenol, Ganciclovir, etc. in use by the patients. They were excluded from further analysis. The remaining 108 metabolites were subjected to statistical analysis. Welch's *t*-test identified 29 metabolites that exhibited significant difference between the Normal-KF and Impaired-KF groups (Fold change > 2, *p* < 0.05). Among these, 9 metabolites were enriched in Normal-KF, and 20 were enriched in Impaired-KF.

Table 2 listed these 29 metabolites along with their *p-*value and fold changes between Normal-KF and Impaired-KF. As expected, creatinine was one of the enriched metabolites in Impaired-KF. Additionally, each metabolite's Pearson correlation coefficient with creatinine, calculated using MS signals from individual samples, was included in Table 2. Supplementary Figure 1 presents box whisker plot comparisons of these metabolites between the Normal-KF and Impaired-KF groups, arranged by ascending *p*-value.

**Table 2:** List of differential metabolites, arranged by *p*-value.

No.	Metabolites	Formula	CAS number	ID confidence level	Pearson correlation coefficient with creatinine	*p-*value normal-KF vs. impaired-KF	Fold change normal-KF/impaired-KF
1	Piperine	C_17_H_19_NO_3_	94-62-2	1	−0.47	1.3 × 10^−4^	46
2	D-Mannitol	C_6_H_14_O_6_	69-65-8	1	0.80	1.5 × 10^−4^	8.7 × 10^−2^
3	meso-Erythritol	C_4_H_10_O_4_	149-32-6	1	0.80	1.5 × 10^−4^	8.7 × 10^−2^
4	N-Acetyl-L-alanine	C_5_H_9_NO_3_	97-69-8	1	0.91	4.1 × 10^−4^	0.46
5	DL-2-Aminocaprylic acid	C_8_H_17_NO_2_	644-90-6	1	−0.57	5.8 × 10^−4^	2.0
6	4-Hydroxyphenylacetic acid	C_8_H_8_O_3_	156-38-7	1	0.38	7.2 × 10^−4^	5.2 × 10^−2^
7	Threonate	C_4_H_8_O_5_	70753-61-6	1	0.76	9.9 × 10^−4^	0.18
8	N-Acetyl-L-Proline	C_7_H_11_NO_3_	68-95-1	1	0.93	1.9 × 10^−3^	5.8 × 10^−2^
9	Indoleacrylic acid	C_11_H_9_NO_2_	1204-06-4	2	0.25	1.9 × 10^−3^	2.4 × 10^−3^
10	N-Acetyl-DL-tryptophan	C_13_H_14_N_2_O_3_	87-32-1	1	0.13	2.7 × 10^−3^	8.4 × 10^−4^
11	(E)-Urocanic acid	C_6_H_6_N_2_O_2_	3465-72-3	1	−0.40	2.7 × 10^−3^	2.1
12	Nε,Nε,Nε-Trimethyllysine	C_9_H_21_N_2_O_2_	55528-53-5	2	0.93	4.7 × 10^−3^	0.47
13	Caffeine	C_8_H_10_N_4_O_2_	58-08-2	1	−0.32	4.8 × 10^−3^	13
14	Creatinine	C_4_H_7_N_3_O	60-27-5	1	1	5.0 × 10^−3^	0.24
15	Theophylline	C_7_H_8_N_4_O_2_	58-55-9	1	−0.37	5.8 × 10^−3^	14
16	Paraxanthine	C_7_H_8_N_4_O_2_	611-59-6	2	−0.40	6.7 × 10^−3^	8.1
17	L-Hexanoylcarnitine	C_13_H_26_NO_4_	22671-29-0	2	0.68	1.1 × 10^−2^	0.27
18	4-Pyridoxic acid	C_8_H_9_NO_4_	82-82-6	2	0.63	1.2 × 10^−2^	4.8 × 10^−2^
19	Indolelactic acid	C_11_H_11_NO_3_	1821-52-9	2	0.52	1.4 × 10^−2^	0.19
20	SAH/S-Adenosyl-L-homocysteine	C_14_H_20_N_6_O_5_S	979-92-0	2	0.80	1.6 × 10^−2^	0.34
21	(±)-Octanoylcarnitine	C_15_H_30_NO_4_	25243-95-2	2	0.78	1.8 × 10^−2^	0.42
22	Quinic acid	C_7_H_12_O_6_	77-95-2	1	0.49	2.0 × 10^−2^	0.20
23	S-Methyl-L-cysteine sulfoxide	C_4_H_9_N O_3_S	32726-14-0	1	−0.46	2.1 × 10^−2^	2.2
24	Butyryl-L-carnitine	C_11_H_22_NO_4_	25576-40-3	2	0.90	2.1 × 10^−2^	0.15
25	L-(+)-Ergothioneine	C_9_H_15_N_3_O_2_S	497-30-3	1	−0.32	2.4 × 10^−2^	4.0
26	L-Theanine	C_7_H_14_N_2_O_3_	3081-61-6	1	0.04	2.5 × 10^−2^	0.34
27	Sucrose	C_12_H_22_O_11_	57-50-1	1	0.19	3.0 × 10^−2^	2.3 × 10^−2^
28	Niacinamide	C_6_H_6_N_2_O	98-92-0	1	−0.24	4.0 × 10^−2^	2.4
29	3-Methylxanthine	C_6_H_6_N_4_O_2_	1076-22-8	1	0.41	4.7 × 10^−2^	0.27

Normal-KF, normal kidney function; Impaired-KF, impaired kidney function.

**Figure 1:**
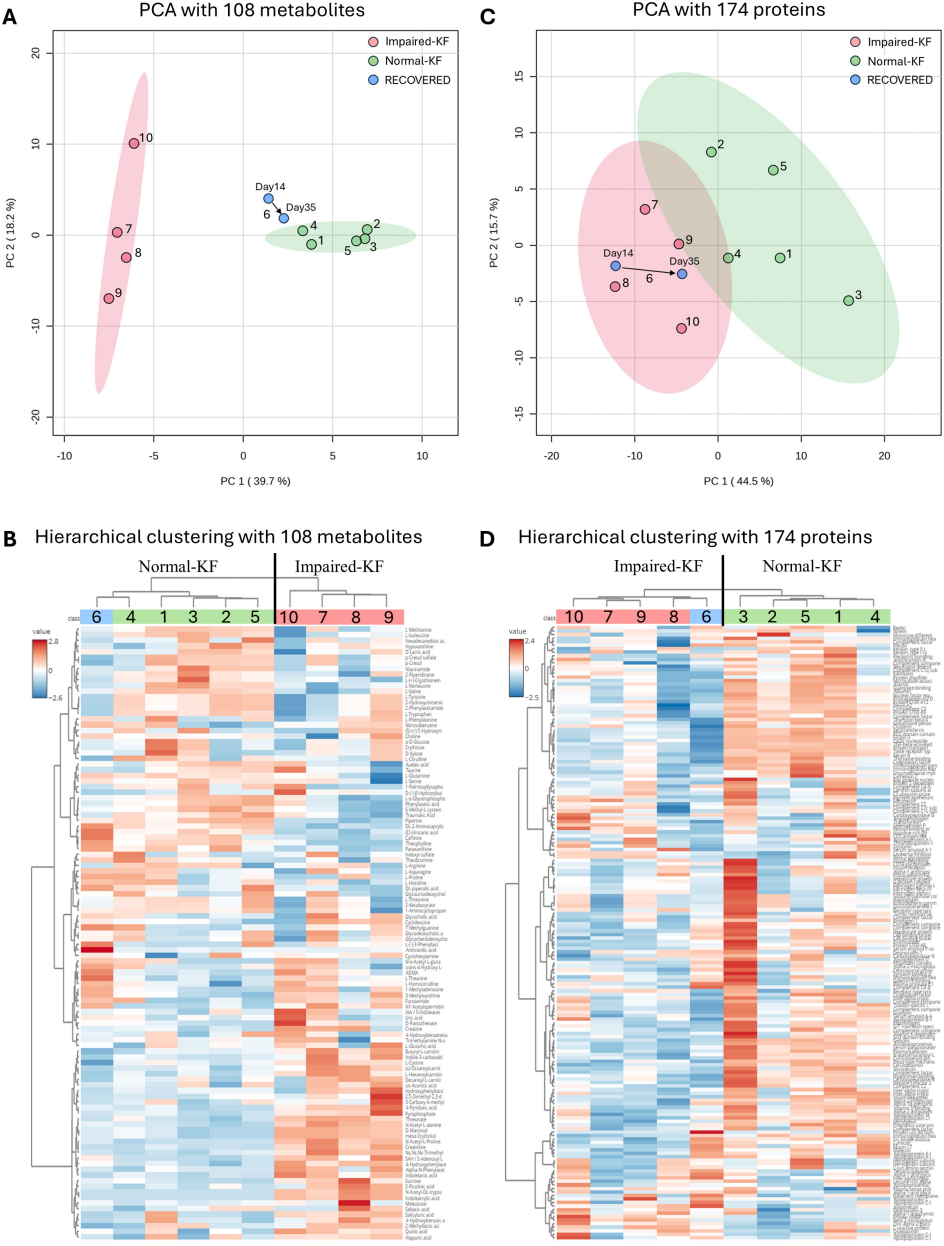
Clustering analysis of metabolites and proteins. **(A,C)** Principal component analysis (PCA) with 108 metabolites **(A)** or 174 proteins **(C)**, with colored regions representing 95% confidence intervals. **(B,D)** Hierarchical clustering analysis with 108 metabolites **(B)** or 174 proteins **(D)**. Normal-KF, normal kidney function; Impaired-KF, impaired kidney function.

Furthermore, PCA revealed clear separation between the Normal-KF and Impaired-KF groups (Figure 1A). Samples from the recovered patient (No. 6) on day 14 and day 35 were positioned near the Normal-KF group, with the day-35 sample trending even closer (Figure 1A). Using the average signal from two samples of the same patients, hierarchical clustering similarly grouped the recovered patient (No. 6) with the Normal-KF group, showing a closer relationship to patient No. 4, consistent with the PCA results (Figure 1B).

### Proteomic analysis

A total of 1,493 unique peptides met the quantification criteria, corresponding to 174 proteins, each represented by 1–193 peptides. Welch's *t*-test identified 45 proteins showing significant difference between the Normal-KF and Impaired-KF groups (Fold change > 1.25, *p* < 0.05). Among these, 43 proteins exhibited higher expression in the Normal-KF group, while only 2 proteins showed higher expression in the Impaired-KF group: β2M and protein AMBP (alpha-1-microglobulin/bikunin precursor) (Table 3). These 45 proteins were analyzed for pathway enrichment using Reactome v90 (https://www.reactome.org) (18, 19). Table 4 presents ten biological pathways potentially affected by impaired kidney function post-LT (False discovery Rate < 0.01). Proteins associated with these ten pathways are indicated in Table 3.

**Table 3:** List of differential proteins, arranged by *p*-value.

No.	Proteins	UniProt number	peptide count	*p*-value Normal-KF vs. Impaired-KF	Fold change Normal-KF/Impaired-KF	Reactome Pathway No.
1	PDZ domain-containing protein 2	O15018	1	2.10 × 10^−4^	1.57	
2	Serotransferrin	P02787	37	9.35 × 10^−4^	1.53	1, 2, 3, 4
3	Antileukoproteinase	P03973	2	1.01 × 10^−3^	1.67	6
4	Beta-2-microglobulin	P61769	3	1.04 × 10^−3^	0.40	6, 9
5	Protein transport protein Sec16A	O15027	1	1.12 × 10^−3^	1.75	
6	Ficolin-3	O75636	1	3.21 × 10^−3^	1.49	6
7	Src kinase-associated phosphoprotein 2	O75563	1	4.10 × 10^−3^	1.32	
8	Apolipoprotein A-II	P02652	4	4.63 × 10^−3^	1.66	1, 2, 10
9	Immunoglobulin heavy constant gamma 2	P01859	2	4.81 × 10^−3^	1.64	6
10	Fetuin-B	Q9UGM5	2	5.28 × 10^−3^	1.64	
11	Taste receptor type 2 member 14	Q9NYV8	1	6.28 × 10^−3^	1.70	
12	Alpha-2-HS-glycoprotein	P02765	13	6.80 × 10^−3^	1.59	1, 2, 3, 4, 6
13	Cyclic nucleotide-gated cation channel beta-3	Q9NQW8	1	7.21 × 10^−3^	1.42	
14	Prostaglandin-H2 D-isomerase	P41222	1	1.09 × 10^−2^	1.28	
15	Ceruloplasmin	P00450	39	1.13 × 10^−2^	1.73	1, 2
16	Hepatocyte growth factor-like protein	P26927	1	1.36 × 10^−2^	1.39	
17	Unconventional myosin-Vb	Q9ULV0	1	1.39 × 10^−2^	1.52	
18	Gelsolin	P06396	20	1.83 × 10^−2^	1.47	6, 9
19	TGF-beta-activated kinase 1 and MAP3K7-binding protein 3	Q8N5C8	1	1.85 × 10^−2^	1.65	6
20	Malectin	Q14165	1	1.93 × 10^−2^	1.28	6
21	Insulin-like growth factor-binding protein complex acid labile subunit	P35858	1	1.93 × 10^−2^	1.33	1
22	Plasma kallikrein	P03952	6	1.99 × 10^−2^	1.31	
23	Immunoglobulin gamma-1 heavy chain	P0DOX5	12	2.37 × 10^−2^	1.84	
24	Protein S100-A9	P06702	1	2.62 × 10^−2^	1.37	6, 8
25	Corticosteroid-binding globulin	P08185	10	2.69 × 10^−2^	1.38	
26	Pregnancy zone protein	P20742	2	2.91 × 10^−2^	1.51	
27	Vitamin D-binding protein	P02774	25	2.93 × 10^−2^	1.31	
28	Mucin-17	Q685J3	1	2.95 × 10^−2^	1.29	6
29	Serum amyloid P-component	P02743	6	3.08 × 10^−2^	1.40	9
30	Apolipoprotein A-I	P02647	17	3.08 × 10^−2^	1.39	1, 2, 3, 4, 5, 7, 9, 10
31	Hyaluronan-binding protein 2	Q14520	1	3.10 × 10^−2^	1.31	
32	Microtubule-associated serine/threonine-protein kinase 2	Q6P0Q8	1	3.13 × 10^−2^	1.35	
33	Alpha-1B-glycoprotein	P04217	16	3.18 × 10^−2^	1.30	3, 4, 6
34	Immunoglobulin kappa constant	P01834	5	3.34 × 10^−2^	1.60	5, 6, 7
35	Thymosin beta-4	P62328	1	3.61 × 10^−2^	2.06	3, 4
36	Heparin cofactor 2	P05546	19	3.63 × 10^−2^	1.52	1, 2
37	Piezo-type mechanosensitive ion channel component 1	Q92508	1	3.67 × 10^−2^	1.30	
38	Serum paraoxonase/arylesterase 1	P27169	11	3.76 × 10^−2^	1.62	
39	Complement factor H	P08603	40	3.77 × 10^−2^	1.35	6
40	Protein AMBP	P02760	12	3.78 × 10^−2^	0.66	5, 7
41	Nuclear factor erythroid 2-related factor 2	Q16236	1	3.90 × 10^−2^	1.33	
42	Albumin	P02768	34	4.05 × 10^−2^	1.33	1, 2, 3, 4, 5, 7, 10
43	Guanylate-binding protein 1	P32455	1	4.23 × 10^−2^	1.28	
44	Apolipoprotein L1	O14791	6	4.38 × 10^−2^	1.50	1, 2, 5, 7
45	Protein S100-A8	P05109	2	4.38 × 10^−2^	1.82	6, 8

Normal-KF, normal kidney function; Impaired-KF, impaired kidney function. Details about Reactome pathway numbers can be found in Table 4.

**Table 4:** Reactome pathway analysis of differential proteins.

No.	Pathway identifier	Pathway name	#Entities found	#Entities total	Entities *p*-value	Entities FDR
1	R-HSA-381426	Regulation of Insulin-like Growth Factor (IGF) transport and uptake by Insulin-like Growth Factor Binding Proteins (IGFBPs)	9	124	9.55 × 10^−10^	2.79 × 10^−7^
2	R-HSA-8957275	Post-translational protein phosphorylation	8	107	7.11 × 10^−9^	1.04 × 10^−6^
3	R-HSA-114608	Platelet degranulation	6	128	9.05 × 10^−6^	8.20 × 10^−4^
4	R-HSA-76005	Response to elevated platelet cytosolic Ca2+	6	133	1.12 × 10^−5^	8.20 × 10^−4^
5	R-HSA-2168880	Scavenging of heme from plasma	5	99	3.78 × 10^−5^	2.19 × 10^−3^
6	R-HSA-168249	Innate Immune System	14	1,187	8.42 × 10^−5^	4.04 × 10^−3^
7	R-HSA-2173782	Binding and Uptake of Ligands by Scavenger Receptors	5	129	1.30 × 10^−4^	5.35 × 10^−3^
8	R-HSA-6799990	Metal sequestration by antimicrobial proteins	2	6	2.51 × 10^−4^	8.35 × 10^−3^
9	R-HSA-977225	Amyloid fiber formation	4	81	2.61 × 10^−4^	8.35 × 10^−3^
10	R-HSA-8963899	Plasma lipoprotein remodeling	3	35	3.34 × 10^−4^	9.68 × 10^−3^

FDR, false discovery rate.

Similar to the metabolomic analysis, we performed protein cluster analysis using all 174 proteins. PCA revealed a separation between the Normal-KF and Impaired-KF groups (Figure 1C), although this separation was less distinct than what was observed for metabolites. Interestingly, for the recovered patient (No. 6), both samples clustered closely with the Impaired-KF group, with the day 35 sample trending towards the Normal-KF group (Figure 1C). In hierarchical clustering, this patient was grouped with the Impaired-KF group, contrasting with the results from metabolomics profiling (Figure 1D).

## Discussion

Our findings revealed distinct metabolomic and proteomic signatures associated with kidney impairment post-LT. Specifically, for the patient whose eGFR returned to normal after LT, the metabolomic profile closely resembled that of patients with Normal-KF, suggesting recovery at the metabolic level. However, the proteomic profile remained aligned with the Impaired-KF group, indicating that underlying changes in protein expression may persist longer despite improved renal filtration. This discrepancy highlights the complex and multi-layered nature of kidney recovery and suggests that metabolic and proteomic pathways may recover at different rates post-LT.

Given these findings, reliance solely on serum creatinine-based eGFR may not provide an accurate and complete picture of kidney recovery or ongoing dysfunction in LT recipients. Incorporating additional measures into routine post-transplant monitoring could provide a more comprehensive assessment of kidney health and enable timely intervention to prevent renal dysfunction progression. Moreover, proteomic analysis may uncover lingering inflammation or structural changes within the kidney that persist despite improved filtration markers, providing insights into long-term recovery trajectories.

Due to the nature of the untargeted LC-MS/MS methodology, the proteins identified in our analysis tend to be abundant in the bloodstream. While many proteins could be of interest, such as NGAL and TIMP-1, their low abundance limited our ability to detect or quantify them in this study. Despite these limitations, our analysis identified 45 differentially expressed proteins, with β2M and alpha-1-microglobulin (encoded by protein AMBP) being enriched in the Impaired-KF group. Both proteins are known to be elevated in patients with kidney disease (20). In contrast, the remaining 43 differential proteins were enriched in the Normal-KF group. These proteins are involved in various biological pathways, including Insulin-like growth factor regulation, post-translational protein phosphorylation, and the innate immune system. Further investigation into these proteins could deepen our understanding of the molecular mechanisms underlying kidney injury and recovery following LT.

Besides creatinine, we identified 28 metabolites that differ significantly between Normal-KF and Impaired-KF. Among these, several metabolites showed a strong positive correlation with creatinine (correlation coefficient > 0.9), such as N-acetyl-alanine, N-acetyl-proline, trimethyl-lysine, and butyryl-carnitine. These metabolites, either individually or in combination with creatinine, may serve as important components of the metabolomic signature associated with Impaired-KF. Their elevated levels could reflect altered metabolic processes such as amino acid metabolism, mitochondrial dysfunction, and disruptions in carnitine-related energy production, which are commonly observed in kidney impairment.

In contrast, some metabolites exhibited a negative correlation with creatinine, suggesting they may play a protective role in kidney health. For instance, compounds such as piperine, ergothioneine, and S-methyl-cysteine-sulfoxide were identified, all of which are recognized for their antioxidant properties and potential health benefits (21–23). Among them, piperine emerged as the most significant metabolite, showing the highest fold change and the smallest *p-*value in our analysis. Previous studies have demonstrated the protective effects of piperine in kidney injury models, including ischemia-reperfusion-induced damage and lead acetate-induced nephrotoxicity (24, 25). Additionally, we observed an enrichment of caffeine and its downstream metabolites, paraxanthine and theophylline, in the Normal-KF group. This finding aligns with research indicating that coffee consumption is associated with a reduced risk of both acute and chronic kidney diseases (26, 27). Future studies focusing on their mechanisms of action, pharmacological effects, and clinical applications could provide valuable insights into novel strategies for kidney injury prevention and treatment.

A key limitation of our study is the small patient cohort. Despite this, our findings reveal distinct metabolomic and proteomic signatures associated with kidney impairment post-LT. Interestingly, for the patient with recovered eGFR, the metabolomic profile aligned closely with the Normal-KF group, whereas the proteomic profile remained closer to the Impaired-KF group. This divergence underscores the importance of a multifaceted approach to post-LT kidney function monitoring. Another limitation of this study is its correlative nature, which prevents us from establishing causality between the identified metabolites, proteins, and kidney function. Given this, our study should be viewed as hypothesis-generating, laying the groundwork for further research to elucidate the roles and functional implications of the differential proteins and metabolites identified in this study.

## Data Availability

The mass spectrometry data have been deposited to the ProteomeXchange Consortium via the PRIDE (28) partner repository with the dataset identifier PXD062924. Other original contributions presented in the study are included in the article/Supplementary Material. Further inquiries can be directed to the corresponding author.

## In memoriam

The group wishes to dedicate this work to the loving memory of Dr. Josh Levitsky. His friendship, and unwavering passion for discovery will leave a lasting legacy.
