# Supplementary material for: Metabolomic and proteomic analyses of renal function after liver transplantation
Source: Front Transplant. 2025 Apr 29;4:1572852. doi: 10.3389/frtra.2025.1572852 (PMC12069452; doi:10.3389/frtra.2025.1572852)
Supplement: Supplementary file 1 [file Image1.pdf]

Supplementary materials

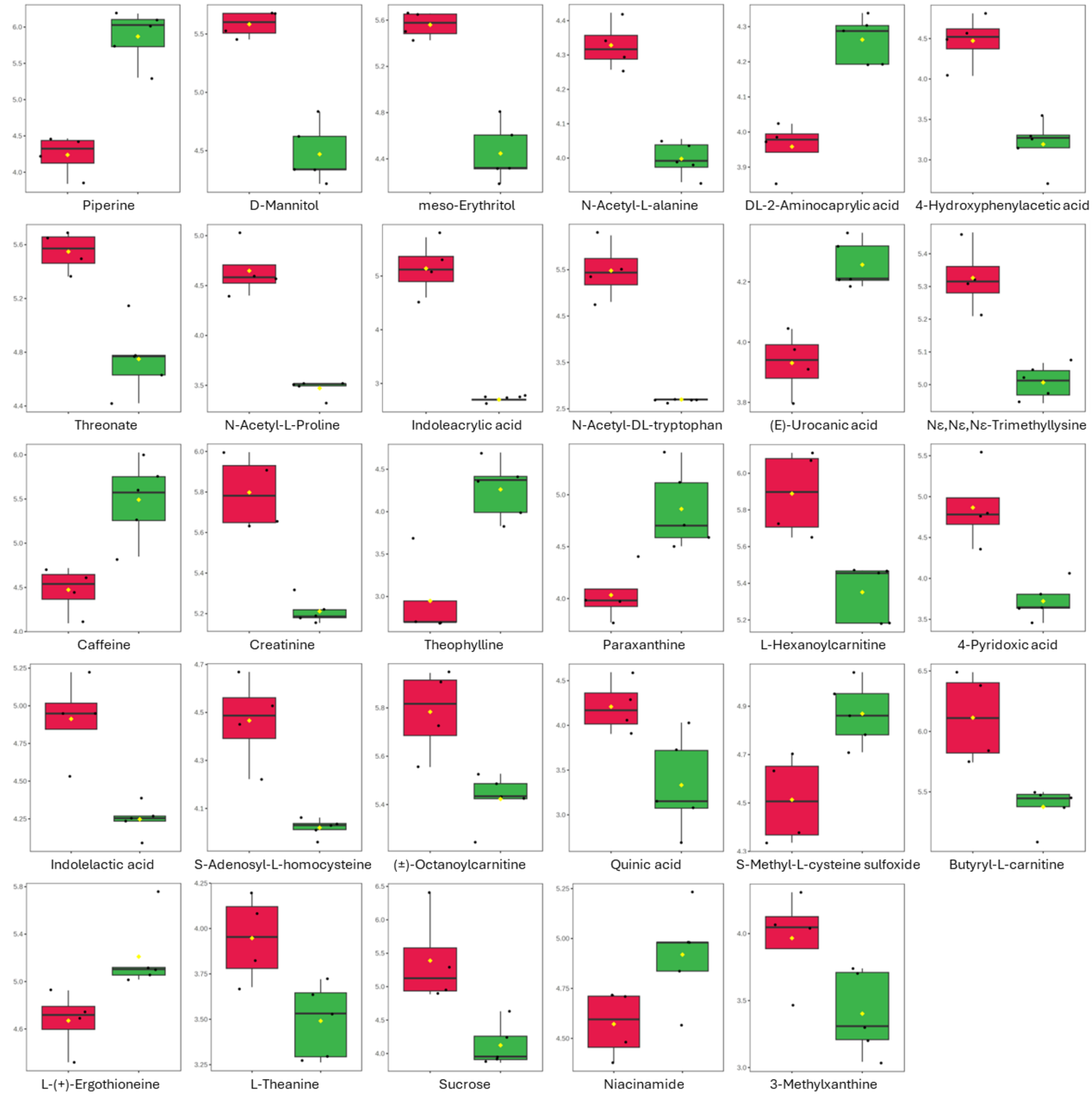

Supplementary Figure 1. Twenty-nine differential metabolites are shown as box-and-whisker plots, arranged by ascending *p*-value. Differences between the impaired kidney function (red) and normal kidney function (green) groups were assessed using Welch's t-test, with significance defined as $p < 0.05$ and fold change > 2. For *p*-values see **Table 2**.
